# *Malva parviflora* Leaves Mucilage: An Eco-Friendly and Sustainable Biopolymer with Antioxidant Properties

**DOI:** 10.3390/polym13234251

**Published:** 2021-12-03

**Authors:** Ans Munir, Fadia S. Youssef, Saiqa Ishtiaq, Sairah H. Kamran, Alaa Sirwi, Safwat A. Ahmed, Mohamed L. Ashour, Sameh S. Elhady

**Affiliations:** 1 Department of Phrmacognosy, College of Pharmacy, University of the Punjab, Lahore 54000, Pakistan; ansmunir92@gmail.com; 2 Department of Pharmacognosy, Faculty of Pharmacy, Ain-Shams University, Cairo 11566, Egypt; fadiayoussef@pharma.asu.edu.eg; 3 Institute of Pharmacy, Faculty of Pharmaceutical Sciences and Allied Health Sciences, Lahore College for Women University, Lahore 54000, Pakistan; sairah.hafeez@lcwu.edu.pk; 4 Department of Natural Products, Faculty of Pharmacy, King Abdulaziz University, Jeddah 21589, Saudi Arabia; asirwi@kau.edu.sa (A.S.); ssahmed@kau.edu.sa (S.S.E.); 5 Department of Pharmacognosy, Faculty of Pharmacy, Suez Canal University, Ismailia 41522, Egypt; safwat_ahmed@pharm.suez.edu.eg; 6 Pharmacy Program, Department of Pharmaceutical Sciences, Batterjee Medical College, Jeddah 21442, Saudi Arabia

**Keywords:** emulsion capacity, FTIR, *Malva parviflora*, natural polymers, physicochemical properties, rheology

## Abstract

*Malva parviflora* L. is an edible and medicinal herb containing mucilaginous cells in its leaves. Mucilage obtained from *M. parviflora* leaves (MLM) was extracted in distilled water (1:10 *w*/*v*) at 70 °C followed by precipitation with alcohol. Preliminary phytochemical tests were performed to assess the purity of the extracted mucilage. Results showed that the yield of mucilage was 7.50%, and it was free from starch, alkaloids, glycosides, saponins, steroids, lipids and heavy metals. MLM had 16.19% carbohydrates, 13.55% proteins and 4.76% amino acids, which indicate its high nutritional value. Physicochemical investigations showed that MLM is neutral and water-soluble, having 5.84% moisture content, 15.60% ash content, 12.33 swelling index, 2.57 g/g water-holding capacity and 2.03 g/g oil-binding capacity. The functional properties, including emulsion capacity, emulsion stability, foaming capacity and stability increased with increased concentrations. Micromeritic properties, such as bulk density, tapped density, Carr’s index, Hausner ratio, and angle of repose, were found to be 0.69 g/cm^3^, 0.84 g/cm^3^, 17.86%, 1.22 and 28.5, respectively. Scanning electron microscopy (SEM) showed that MLM is an amorphous powder possessing particles of varying size and shape; meanwhile, rheological studies revealed the pseudoplastic behavior of MLM. The thermal transition process of MLM revealed by a differential scanning calorimetry (DSC) thermogram, occurring at a reasonable enthalpy change (∆H), reflects its good thermal stability. The presence of functional groups characteristic of polysaccharides was ascertained by the infrared (IR) and gas chromatography/mass spectrometry (GC/MS) analyses. GC revealed the presence of five neutral monosaccharides; namely, galactose, rhamnose, arabinose, glucose and mannose, showing 51.09, 10.24, 8.90, 1.80 and 0.90 mg/g of MLM, respectively. Meanwhile, galacturonic acid is the only detected acidic monosaccharide, forming 15.06 mg/g of MLM. It showed noticeable antioxidant activity against the DPPH (1,1-diphenyl-2-picrylhydrazyl) radical with an IC_50_ value of 154.27 µg/mL. It also prevented oxidative damage to DNA caused by the Fenton reagent, as visualized in gel documentation system. The sun protection factor was found to be 10.93 ± 0.15 at 400 µg/mL. Thus, MLM can be used in food, cosmetic and pharmaceutical industry and as a therapeutic agent due to its unique properties.

## 1. Introduction

Mucilage is a plant hydrocolloid that, upon hydrolysis, yields a mixture of sugars and uronic acids [1]. Structurally, mucilage is a complex of polymeric polysaccharides; in addition, they also contain glycoproteins and various bioactive components, represented by tannins, alkaloids and steroids. Plant-derived mucilage can be obtained from the special mucilage cells of different plant parts. Today, due to bio-incompatibility, toxicity and carcinogenicity of synthetic polymers, greater attention was given to plant-based, naturally derived biopolymers (mucilage, gums, etc.) as key ingredients in the formulation of sustainable, ecofriendly and cost effective products. Moreover, a greater interest was given to natural polymers aiming to encourage “green” materials from “green” chemistry and technologies. Their unique biological properties as well as their chemical flexibility make them the material of choice for efficient drug delivery [2].

Chemically, mucilages are polysaccharides extensively used in pharmaceutical and medicinal applications, due to their diverse physicochemical properties [3]. Pharmaceutically, they are employed as tablet binders and disintegrants, and as suspending, emulsifying, thickening, gelling, stabilizing and sustaining agents in tablets, in addition to their utilization as protective colloids in suspension [4]. Mucilages obtained from *Abelmoschus*, *Aloe*, *Asario*, *Bavchi*, *Fenugreek*, *Hibiscus*, *Ispagol*, *Ocimum* seed, *Satavari* and *Cactus* plants are examples of pharmaceutically important mucilages [4]. Traditionally, they are used to treat diabetes, enhance wound healing, boost immunity and prevent cancer. They are also used as antitussive, antispasmodic, antitoxin and angiotensin-converting enzyme inhibitory agents [5,6].

Mucilages possess antioxidant potential, which lower the aging process and hence can be used in cosmetics. Mucilage has high demand for water purification, due to high water absorption capacity. In the food industry, there is a growing interest in processed foods that contain mucilage, such as thickening, gel formation and emulsifying agents. It is also used as a stabilizer in the dairy industry, such as in ice cream, yogurt and flavored milk. In addition, mucilage-based hydrocolloids have been used in the food industry to offer textural functionality, such as fruit filling, water binding in meat products, milk-based beverages, desserts and jams. Mucilages have excessive use in ink, glue and adhesive industries [2,7].

Nowadays, intensive research is ongoing on plant-derived mucilage-based coating and films. In addition, mucilage is used to develop nanofibers that are used in textile industry fabrication. In coming years, there will be a significant rise in mucilage demand in food, cosmetics and pharmaceutical industries. However, a significant amount of mucilage for industries is not available and statistical consumption of mucilage is not reported. Thus, it was felt mandatory to start the research and commercialization of more natural polymers for drug delivery systems. Therefore, polymer and pharmaceutical scientists are interested in characterizing more natural polymers to be transferred to the market, where the study and consumption of natural polymer for drug delivery (naturapolyceutics) is essential for the present and the future [8,9].

*Malva parviflora* L. is a highly popular species in the genus *Malva* family Malvaceae, commonly known as Cheeseweed, or small whorl mallow, and named in Pakistan by Sonchal. Its leaves possess a pleasant taste and are used as vegetables and salad. Decoction of the whole plant is used to treat cold, fever and cough in folk medicine; meanwhile, a poultice made from the leaves is also used to treat wounds, swellings, abdominal pain, gastritis, constipation, diarrhea, anthelmintic, hair loss, scorpion sting, cataplasm, bladder ulcer, renal inflammation, diuretic, ocular disease and profuse menstruation [10,11].

Furthermore, *M. parviflora* was reported to possess many pharmacological activities comprising antibacterial, antifungal, antidiabetic, antioxidant, anti-inflammatory, neuroprotective, hepatoprotective, anti-irritant, wound healing, analgesic and antiulcerogenic activities [12]. These pronounced activities are mainly attributed to its richness with secondary metabolites, such as flavonoids and phenolic acids [13]. It is noteworthy to highlight that mucilage is one of the major constituents prevailing in genus *Malva*, owing to the presence of mucilaginous cells, which play a crucial role in its therapeutic activities [14,15,16].

Although mucilaginous cells were previously reported in the leaves of *M. parviflora,* nothing was found in the literature regarding its extraction and potential use as a source of eco-friendly and sustainable biopolymer with multiple medicinal values. Thus, in this study, we aimed to isolate mucilage from *M. parviflora* leaves and discover its pharmaceutical and biological properties. Mucilage was extracted in distilled water (1:10 *w*/*v*) at 70 °C followed by precipitation with alcohol. Preliminary phytochemical tests were performed to assess the purity of the extracted mucilage. Various physicochemical, functional and micromeritic properties of powdered mucilage were also investigated. Infrared and gas chromatography, coupled with mass spectrometry analyses, total phenolic and total flavonoid content were also performed. In vitro antioxidant activity was studied by using the 1,1-diphenyl-2-picrylhydrazyl (DPPH*) radical scavenging capacity assay; furthermore, in vitro DNA damage protection and in vitro solar protection factor (SPF) were also determined.

## 2. Materials and Methods

### 2.1. Plant Material 

*Malva parviflora* L. (Malvaceae) was collected from Begum Kot near Shahdra, Lahore, Punjab, Pakistan, in February 2018. Authentication was kindly performed by Dr. Zaheer-ud-din Khan, Plant Taxonomist, Department of Botany, Government College University, Lahore. A specimen with voucher number GC.Herb.Bot.3533 was deposited in the Herbarium of the Department of Botany, GC University, Lahore, Pakistan. Leaves were separated and washed with tap water in order to remove any adhering materials. They were shade-dried and powdered by a grinder that was consequently exposed to extraction.

### 2.2. Extraction of Mucilage

Mucilage was extracted by the method previously described by Malviya [17], with slight modifications. Briefly, dried powdered leaves were soaked in warm distilled water in a ratio of 1:10 (*w*/*v*) for 4 h. Then, heating was performed in a water bath with continuous stirring at 70 °C for 1 h and cooled at room temperature for 1 h. The thick mixture was squeezed in a muslin cloth to remove the residue from the filtrate. The viscous filtrate was concentrated under reduced pressure at 60–70 °C using a rotary evaporator (Laborota 4002, Heidolph, Schwabach, Germany). Mucilage was precipitated from the viscous concentrate by the addition of two volumes of absolute alcohol. Precipitated mucilage was re-dissolved in distilled water, centrifuged and the supernatant was collected. Alcohol was added in supernatant to re-precipitate the mucilage. The precipitation step was repeated to improve the quality of mucilage. Precipitated mucilage was separated, washed with absolute alcohol and dried at 45 °C, and powdered and passed through sieve no. 80. The powdered mucilage was labeled as MLM and stored in a desiccator until further use.

### 2.3. Preliminary Confirmative Tests for Mucilage 

The presence of mucilage was confirmed by performing Molisch’s test and ruthenium red test; meanwhile, the iodine test was done to check the presence of starch [1]. 

### 2.4. Determination of the Yield Percentage

The percentage of mucilage yield was calculated by comparing the weight of dried extracted mucilage with the weight of the initial sample by using the following equation [18].

$$\%Yield=\frac{wt.\;\;of\;dried\;mucilage\;obtained\;}{wt.\;of\;sample\;taken}\times 100\;$$

### 2.5. Determination of the Purity of Mucilage

Qualitative phytochemical tests were performed to check the presence of impurities, such as reducing sugars, proteins, amino acids, lipids, tannins, alkaloids, flavonoids, steroids, glycosides, fats and oils [19].

### 2.6. Elemental Analysis Using Atomic Absorption Spectroscopy 

MLM was digested using dry ash technique followed by the analysis of Cu, Pb, Ni, Cr, Fe, Zn and Cd using the atomic absorption flame spectrophotometer (AA7000, Shimadzu, Kyoto, Japan) [20].

### 2.7. Determination of the Nutritional Value of Mucilage

#### 2.7.1. Estimation of Total Carbohydrates 

Total carbohydrate content was estimated by the anthrone method, where various glucose concentrations were used as a standard for constructing the calibration curve [21].

#### 2.7.2. Estimation of Total Proteins

Total proteins were determined employing the method previously reported by Lowry et al., in which bovine serum albumin (BSA) was used as a standard to establish the calibration curve [22].

#### 2.7.3. Estimation of Total Free Amino Acids 

Total free amino acids were estimated employing the method previously described by Sadasivam, where L-leucine was used as a standard to form a calibration curve [21]. In this method, 80% ethanol (10 mL) was added into a known amount of MLM (100 mg) and exposed to vortex for 1 min. The mixture was transferred to centrifuge tube, centrifuged at 3000 rpm and the supernatant was collected. A total of 0.1 mL of the supernatant was taken and made up the volume up to 1 mL with distilled water. The reagent blank was prepared in the same way by taking 0.1 mL of 80% ethanol. A total of 1 mL of ninhydrin reagent was added in all tubes and heated in a boiling water bath for 20 min. A total of 5 mL of distilled water were added in all test tubes and the absorbance was measured at 570 nm using a spectrophotometer.

### 2.8. Determination of the Physicochemical Properties of the Mucilage

#### 2.8.1. Evaluation of the Organoleptic Properties of the Mucilage 

Organoleptic properties, such as color, odor, taste, shape, touch and texture were evaluated using sense organs comprising eye, nose, tongue, skin, and ear [23].

#### 2.8.2. Evaluation of the Solubility of the Mucilage 

The solubility of extracted mucilage was checked in different solvents, such as water, hot water, 1 M NaOH, 1 M HCl and 1 M citric acid, and organic solvents, such as methanol, ethanol, acetone, petroleum ether and carbon tetrachloride [24]. 

#### 2.8.3. Determination of the pH of the Mucilage 

A pH of 1% *w*/*v* aqueous mucilage solution was determined using a pH meter [25].

#### 2.8.4. Determination of the Moisture Content of the Mucilage

The moisture content of the mucilage was determined using a moisture analyzer (MA35, Sartorius, Gottingen, Germany) [24]. 

#### 2.8.5. Determination of the Ash Content of the Mucilage

Total ash content, water-soluble and acid insoluble ash was determined by a previously reported procedure by Schuck et al. [26].

#### 2.8.6. Determination of the Swelling Index of the Mucilage 

The swelling index was determined using the method previously described by Killedar et al. [27]. Briefly, 1 g of dried powdered mucilage was taken in a 100 mL stopper graduated cylinder. The initial bulk volume was measured, and then 1 mL of alcohol was added, followed by a sufficient quantity of distilled water to form 100 mL of uniform dispersion. Consequently, the cylinder was agitated every 10 min for the first 1 h and kept aside for 3 h. The agitation was repeated at 3 h intervals, and finally, the volume was measured after 24 h. Three readings were taken simultaneously, and the average of the results was reported [27].

#### 2.8.7. Determination of the Water-Holding Capacity of the Mucilage 

Water-holding capacity (WHC) was determined using a method previously reported by Segura-Campos et al. [28]. An empty tube of 15 mL was weighed, then 1 g of powdered mucilage was put in the falcon tube and re-weighed, followed by the addition of 10 mL of distilled water, where the mixture was exposed to the vortex (Seoul in Biosciences, SL V-6, Seoul, Korea) for 5 min. Centrifugation was performed at 3000 rpm (2-16KC, Sigma, Taufkirchen, Germany) for 30 min. Then, the supernatant was removed carefully. Finally, the falcon tube was re-weighed along with the residue. The water-holding capacity of mucilage was calculated using the following equation:

$$WHC=\frac{weight\;of\;wet\;sample-weight\;of\;dry\;sample}{\;weight\;of\;the\;dry\;sample}.\;$$

#### 2.8.8. Determination of the Oil Binding Capacity of the Mucilage 

Oil binding capacity was determined using the method previously reported by Segura-Campos et al. [28]. An empty tube of 15 mL was weighed, then 1 g of powdered mucilage was put in the falcon tube and re-weighed, followed by the addition of 10 mL of sunflower oil where the mixture was exposed to the vortex (SL V-6, Seoul in Biosciences, Seongnam, Korea) for 5 min. Centrifugation was performed at 3000 rpm (2-16KC, Sigma, Taufkirchen, Germany) for 30 min. Then, the supernatant was removed carefully. Finally, the falcon tube was re-weighed along with the residue. The water-holding capacity of mucilage was calculated using the following equation:

$$Oil\;binding\;capacity=\frac{weight\;of\;wet\;sample-weight\;of\;dry\;sample}{\;weight\;of\;the\;dry\;sample\;}\;.\;$$

### 2.9. Determination of the Rheological Properties of the Mucilage

*M. parviflora leaves mucilage* (MLM) powder was dispersed in distilled water to prepare 2.5% (*w*/*v*). Then it was placed for 12 h at 4 °C for complete hydration. The rheological property of this dispersion was observed using a rheometer (AR 1500ex, TA instruments, New Castle, DE, USA). The rheograms of this dispersion were taken at room temperature (25 °C) using a rheometer (AR 1500ex, TA instruments, New Castle, DE, USA) with cone and plate geometry. The shear rate was uniformly increased from 0–1000 s^−1^ over 1 min and then decreased to zero over 1 min at room temperature [29]. 

### 2.10. Evaluation of the Functional Properties of the Mucilage 

#### 2.10.1. Determination of Emulsion Capacity 

The sample was accurately weighed and dissolved in 10 mL of distilled water, and then 10 mL of refined commercial sunflower oil was added. In dispersion, the concentration (*w*/*v*) of mucilage was 0.20, 0.40, 0.60, 0.80 and 1%. Each sample was placed on a magnetic stirrer for 30 min. After that, all the emulsions were subjected to centrifugation at 800 rpm for 15 min. The emulsion capacity was calculated using the following equation [24]:

$$\;\;\;\;\;\;\;\;\;\;\;\;\;\;\;\;\;\;\;\;\;\;\;\;\;\;\;\;\;\;\;\;\;\;\;\;\;\;\;\;\;Emulsion\;capacity=\frac{initial\;emulsion\;volume\;}{total\;volume}\times 100.\;$$

#### 2.10.2. Determination of Emulsion Stability 

Emulsion stability of the prepared mucilage emulsion was determined by heating in a water bath at 80 °C for 30 min. After that, all the emulsions were subjected to centrifugation at 800 rpm for 15 min. Emulsion stability was calculated using the following equation [24]:

$$Emulsion\;stability=\frac{final\;emulsion\;volume}{initial\;emulsion\;volume}\times 100.\;$$

#### 2.10.3. Determination of the Foaming Capacity 

Foaming capacity was determined by the method previously described by Gemede et al. [30] with slight modifications. Briefly, 50 mL solution of mucilage in distilled water at concentrations of 0.2, 0.4, 0.6, 0.8 and 1% (*w*/*v*) were blended in a blender and transferred into a graduated cylinder. The volume was noted, and the foaming capacity was calculated using the following equation:

$$Foaming\;capacity=\frac{FV\;-\;IV}{IV}\times 100.\;$$

*FV*: final-volume; *IV*: initial-volume. 

#### 2.10.4. Determination of the Foaming Stability 

Foaming stability was determined by the method previously described by Gemede et al. [30] with slight modifications. Briefly, 50 mL solution of mucilage in distilled water at concentrations of 0.2, 0.4, 0.6, 0.8 and 1% (*w*/*v*) were blended in a blender and transferred into a graduated cylinder. The volume was noted after 30 min and the foaming stability was calculated using the following equation:

$$Foaming\;stability=\frac{FV\;-\;IV}{IV}\;\times \;100$$

*FV*: final foaming volume; *IV*: initial foaming volume.

### 2.11. Determination of the Micromeritic Properties of the Mucilage 

#### 2.11.1. Determination of the Bulk Density 

The bulk density of mucilage is measured by transferring an accurately weighed powder to a graduated cylinder, and then the bulk volume is noted. The bulk density was calculated by dividing the weight of powder by its bulk volume [31]:

$$Bulk\;density=\frac{weight\;of\;mucilage}{\;bulk\;volume\;}\;$$

#### 2.11.2. Determination of the Tapped Density 

Tapped density was measured by mechanically tapping a graduated cylinder containing accurately weight mucilage powder until the constant volume was observed. The tapped density was calculated by dividing the weight of powder by its tapped volume [31]:

$$Tapped\;density=\frac{weight\;of\;mucilage}{\;tapped\;volume\;}\;$$

#### 2.11.3. Determination of Carr’s Index and Hausner Ratio 

Carr’s or compressibility index and Hausner ratio were calculated using the following formula [32]:

$$Carr’s\;index=\frac{tapped\;density-bulk\;density\;}{tapped\;density}\times 100.\;\;Hausner\;ratio=\frac{tapped\;density\;}{bulk\;density\;}.$$

#### 2.11.4. Determination of Angle of Repose 

The angle of repose is used to evaluate the flow property of powder, where a funnel with the end of the stem cut perpendicular to its axis of symmetry was fixed at a height above the graph paper and placed on a flat horizontal surface. The powder was carefully poured through the funnel until the apex of the conical pile just touched the tip of the funnel. The radius (*r*) of the base of the pile and height (*h*) of the pile was determined. The angle of repose (θ) was calculated using the following equation [32]:

$$\theta \;=\tan^{-1}\frac{h}{r}.\;$$

### 2.12. Morphological Analysis Using Scanning Electron Microscopy

Morphological analysis of powdered MLM surface was performed at different magnifications using a scanning electron microscope (SEM) (JSM-6480LV, JEOL, Tokyo, Japan). Samples were mounted on aluminum stubs, coated with a thin layer of gold and subjected to SEM analysis. The morphological analysis was performed to investigate the particle size, shape and form of the particles [33].

### 2.13. Thermal Analysis Using Differential Scanning Calorimetry 

Differential scanning calorimetry (DSC) was used to study the thermal stability of mucilage. A total of 14 mg of MLM were placed into an aluminum pan and sealed. The measurements were made using DSC Q2000 v24.11 build 124 differential scanning calorimeter, where heating occurs at a rate of 20 °C/min with a temperature range from 0 °C to 300 °C, under a nitrogen atmosphere. Nitrogen gas purging was maintained at 20 mL/min. Meanwhile, an empty aluminum pan was taken as a reference during the analysis [34].

### 2.14. Phytochemical Analysis of the Mucilage

#### 2.14.1. FTIR Spectroscopy Analysis

Attenuated total reflectance (ATR) FTIR spectrophotometric analysis was used to identify the functional group present in MLM powder. An ATR accessory equipped with diamond crystal (Cary 630, Agilent Technologies, Santa Clara, CA, USA) was used for sampling. The data were collected in absorbance (A) mode at frequency regions of 4000–650 cm^−1^ at a resolution of 4 cm^−1^ [35].

#### 2.14.2. Gas Chromatography Coupled with Mass Spectrometry Analysis

Monosaccharide composition of MLM was determined by using a modified GC-MS analytical procedure developed by Xia et al. [36], which is based upon trimethylsilyl dithioacetal (TMSD) derivatization. Briefly, 6 mg of MLM powder were hydrolyzed using 3 mL of 2 M TFA at 110 °C for 2 h in a sealed reaction vessel and nitrogen atmosphere. A mixture of ethanethiol and TFA (2:1, 20 µL) was added in standards or samples and then the reaction vessels were tightly closed with a screw cap. The residue in vessels was dissolved by softly swirling and the resulting solution was kept for 10 min at 25 °C. For TMSD derivatization pyridine (100 µL), 68 µL of hexamethyl disilazane (HMDS) and 22 µL of trimethylchlorsilane (TMCS) were added in natural dried residue. The resulting mixture was heated in water bath at 70 °C for 30 min. Then, suitable amounts of water and chloroform were added for liquid–liquid extraction. The chloroform fraction was filtered through membrane filter (0.22 µm) for GC MS analysis. A total of 1 µL of the sample was injected to the Agilent 7890A gas chromatography coupled to the Agilent 5975C mass spectrometer and the analysis was performed on a column HP-5MS using the following temperature range: 80 °C for 0 min, 80–190 °C at 2.5 °C/min, 190–252 °C at 2 °C/min, 252–300 °C at 25 °C/min and 300–310 °C at 25 °C/min, and held for 15 min. Mass spectra was recorded using a total ion chromatogram (TIC) mode and interpreted using NIST 5 software. 

#### 2.14.3. Estimation of Total Flavonoid Content 

Total flavonoid content was determined using the method previously described by Marinova et al. [37] with slight modifications. Briefly, the MLM sample was prepared in such a way that each sample contains 100 mg/mL. About 1 mL of MLM was mixed with 4 mL of distilled water and 0.3 mL of 5% NaNO_2_. After 5 min, 0.6 mL of 10% AlCl_3_ was added and the mixture incubated at room temperature for five minutes, followed by the addition of 2 mL of 1M NaOH. The volume of mixture was made up to 10 mL by distilled water. The absorbance was taken at 510 nm against the blank. For the calibration curve, rutin was used with a concentration range of 10–100 µg/mL (Absorbance = 0.0079 rutin µg − 0.0267, R^2^ = 0.996).

#### 2.14.4. Estimation of Total Phenolic Content 

Total phenolic content was determined using the method previously described by Saeed et al. [38] with slight modifications. Briefly, the MLM aqueous solution was prepared in such a way that it contained 1 mg/mL. The sample solution (1 mL) and Folin–Ciocalteu’s phenol reagent (Central drug house, New Delhi, India) (1 mL) were mixed in 25 mL volumetric flask. After 5 min, 10 mL of 7% Na_2_CO_3_ solution were added to the mixture and the volume was made up to the mark in each flask by adding distilled water. The mixture was incubated for 90 min at room temperature. After incubation, the absorbance was determined against blank at 750 nm with UV-visible spectrophotometer. Gallic acid was used to construct the calibration curve used with a concentration range of 10–100 µg/mL (Absorbance = 0.0041 gallic acid µg + 0.0212, R^2^ = 0.995).

### 2.15. In Vitro Biological Evaluation of the Mucilage

#### 2.15.1. In Vitro Antioxidant Activity Assessment Using 1,1-Diphenyl-2-picrylhydrazyl (DPPH*) Radical Scavenging Capacity Assay

The mucilage was dissolved in distilled water using different concentrations from 10–1000 µg/mL. DPPH solution (0.1 mM) was prepared using ethanol as a solvent, then, 1 mL of the sample solution was mixed with 1 mL of DPPH. After incubation for 30 min in the dark at room temperature, the absorbance was measured at 517 nm. The scavenging activity of DPPH radical was calculated using the following equation [39,40,41]:

$$Scavenging\;ability\;\left(\%\right)=\left(1-\left(\frac{A_{1}}{A_{0}}\right)\right)\times 100\;$$

*A*_1_ and *A*_0_ are the absorbance of the sample and control (without sample), respectively.

#### 2.15.2. In Vitro Determination of DNA Damage Protective Potential of the Mucilage 

The DNA damage protective activity of MLM was evaluated at three different concentrations (50, 100 and 200 µg/mL), using the previously reported method by Kaur et al. with slight modifications [42]. Briefly, DNA was isolated from human blood using a DNA isolation kit (DNA mini kit 50, Qiagen, Venlo, The Netherland) and was then quantified using nanodrop technology (Nanodrop 2000m Thermo Fisher, Waltham, MA, USA). DNA damage was induced by using Fenton reagent, which is freshly prepared by mixing equal volumes of 80 mM FeCl_3_ (BDH Chemicals Limited, Poole, UK), 50 mM ascorbic acid (Sigma–Aldrich Chemie GmbH, Taufkirchen, Germany) and 30 mM hydrogen peroxide (Merck AG, Darmstadt, Germany). Samples were prepared in such a way that the total volume of the mixture was 20 µL. Six groups were prepared as follows: Group A: 4 µL DNA + 16 µL deionized water (DW); Group B: 3 µL Fenton reagent (FR) +17 µL DW; Group C: 4 µL DNA + 3 µL FR + 13 µL DW; Group D: 4 µL DNA + 4 µL MLM (50 µg/mL) + 3 µL FR + 9 µL DW; Group E: 4 µL DNA + 4 µL MLM (100 µg/mL) + 3 µL FR + 9 µL DW and Group F: 4 µL DNA + 4 µL MLM (200 µg/mL) + 3 µL FR + 9 µL DW. All the above groups were incubated at 37 °C for 30 min. After incubation, 5 µL of bromophenol dye was added to each sample. For gel electrophoresis 1X TAE (40 mM Tris-acetate, 1 mM EDTA) buffer was used as a running buffer. A total of 10 µL sample from each group was loaded in each well of agarose gel (1%), and then the gel was run at 100 V for 1 h. Finally, the gel was observed in the gel documentation system (Gene genius, Syngene, Cambridge, UK) to get the final results.

#### 2.15.3. In Vitro Evaluation of Sun Protection Factor of the Mucilage

Sun protection factor (SPF) was determined using a previously reported method by Suva [43] with slight modifications. MLM was dissolved in distilled water to get solutions with concentrations of 200 and 400 µg/mL. Then, spectrophotometer readings of these solutions were measured at wavelengths ranging from 290 to 320 nm at 5 nm intervals, where SPF for mucilage was calculated from the following equation:

$$SPF=CF\times \sum_{290}^{320}EE\times I\times Abs$$

*EE* (I) Erythemal effect spectrum; *I* (I)—solar intensity spectrum; *Abs*—Absorbance of sunscreen product; *CF*—correction factor (=10); the values of *EE* × *I* are constant and are predetermined.

### 2.16. Statistical Analysis

Statistical analysis was conducted using Microsoft excel (2010). All the experiments were conducted as triplicates and expressed as mean ± standard deviation.

## 3. Results and Discussion 

Various methods were adopted to extract mucilage, comprising the precipitation method, ultrasound-assisted extraction, microwave-assisted extraction and enzyme-assisted extraction. MLM was extracted using the alcohol precipitation method, as it is the most common, inexpensive and easy method for extracting mucilage [44]. 

### 3.1. Determination of the Yield Percentage and Purity of Mucilage 

*M. parviflora leaves mucilage* (MLM) yields 7.50 ± 0.20% (*w*/*w*); meanwhile, it stained pink with ruthenium red and gave a positive result with Molisch’s test. Furthermore, it was free from starch, alkaloids, glycosides, saponins, lipids, steroids and heavy metals. Heavy metals have highly toxic and mutagenic effects even at a low concentrations, where lead, cadmium and chromium are the major cause of metallic toxicity [45]. Thus, MLM was subjected to elemental analysis using the atomic absorption spectroscopy technique. The results revealed that it was almost free from chromium, lead, cadmium and nickel and, in particular, did not have copper, zinc and iron.

### 3.2. Determination of the Nutritional Value of Mucilage

Results obtained from the nutritional value analysis of MLM revealed that carbohydrates, proteins and amino acids were abundantly present in *M. parviflora leaves mucilage*. This was evidenced by the presence of 16.19 ± 0.70% carbohydrates, 13.55 ± 0.44% proteins and 4.76 ± 0.56% amino acids, ensuring the nutritional value. This analysis was repeated in triplicates, where the results are expressed as mean ± standard deviation. 

### 3.3. Determination of the Physicochemical Properties of the Mucilage

Mucilage, when used as an excipient, can change the color, odor and taste of pharmaceutical formulations; therefore, organoleptic evaluation of mucilages was carried out to assess the effect of MLM on the color, odor and taste on pharmaceutical products. Organoleptic examination of MLM revealed that it exhibited brown color, characteristic odor and bland taste with irregular shape, with hard and a rough to touch texture. Pharmaceutical applications of mucilage highly rely upon its physicochemical properties, illustrated in Table 1 for MLM. Solubility is a key factor to sustain the efficiency in biopolymer development, where high solubility improves the appearance and texture of the formulation. MLM solubility behavior was almost following those previously reported for already extracted mucilages [23,46,47]. It displayed solubility in cold and hot water and was less soluble in 1M NaOH, HCl, citric acid and NaCl, and insoluble in all other organic solvents. Concerning the pH value of MLM, it is considered neutral, displaying a pH value of 6.94. It is noteworthy to mention that pH plays an important role in determining the suitability of the excipient in preparation, where the physiological activity and stability of the formulation depend on the pH of the excipient. In addition, pH is also crucial in the process of flocculation and coagulation. Neutral polymers are ideal for water treatment, with high salinity and water hardness [48,49], and additionally, it does not irritate the mucous membrane and gastrointestinal tract when ingested orally. Thus, MLM, being neutral, can be used safely as an excipient in pharmaceutical formulation.

Regarding the moisture content of MLM, it showed 5.84% moisture, which perfectly lies within the pharmacopoeial limit (≤15%) for natural gums and mucilages [50]. Physical, chemical and microbiological properties of excipient and active pharmaceutical ingredients mainly depend on moisture content. Higher moisture content may cause various problems, such as poor powder flow, increased microbial contamination and instability of formulation. Furthermore, total ash content determines the quality and nutritional value of food products. It determines the whole residual material that remains after burning, comprising physiological and non-physiological ash, including extraneous matters, represented by sand and soil [50,51].

Meanwhile, acid-insoluble ash defines the remaining insoluble matter formed after boiling the total ash using dilute hydrochloric acid and followed by ignition. The acid-insoluble ash determines the quantity of silica present, especially sand and siliceous earth. It is often of higher value than the total ash in detecting earthy matter that adheres to the drug. The total ash content for MLM was found to be 15.60%, which is higher than commercially available gums, comprising xanthan gum (1.5%), gum Arabica (1.2%) and Guar gum (11.9%) [52]. Meanwhile, it showed 12.03% water-soluble ash and low acid-insoluble ash to be estimated by 0.87%, which in turn, verifies its purity and high nutritional value. Additionally, MLM displayed a considerable swelling index estimated by 12.33, where a good swelling index is an indication that the polymer can be used as a binder, disintegrant and an in-control release in the drug delivery system. It is also a prediction of the presence of the hydroxyl group, galactose moiety, and ascertains the purity of mucilage [44]. In addition, MLM displayed high water-holding capacity and oil-binding capacity, estimated by 2.57% and 2.03% (g/g), respectively. The water-holding capacity and oil-binding capacity play an important role in maintaining the food texture. High-oil binding capacity reduces the loss of oil and flavor from the food [53].

### 3.4. Determination of the Rheological Properties of the Mucilage 

**Figure 1:**
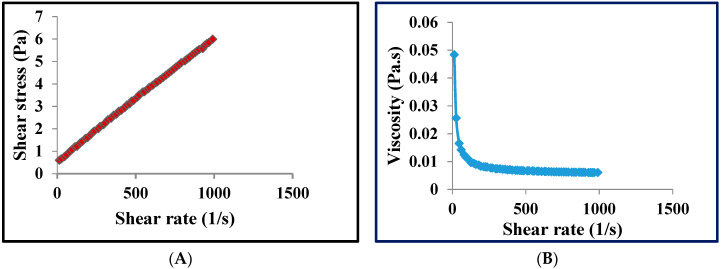
Rheogram of M. *parviflora mucilage* obtained from the leaves (MLM) showing the relationship between shear rate and shear stress (**A**) and the relationship between shear rate and viscosity (**B**). *Pa* = *Pascal*—*unit of shear stress whereas Pa. s*—*Pascal-second unit of viscosity*.

Rheological studies are usually performed to determine the behavior of polymer under stress, where the pseudoplastic behavior of polymers finds considerable application in the food industry. This makes them easily swallowed while eating. In addition, the pseudoplastic behavior of polymers helps in industrial operation comprising mixing and pumping [54]. Rheological studies showed that MLM had shear-thinning behavior or pseudoplastic behavior, a non-Newtonian attitude of fluids, where the viscosity decreases by increasing the shear stress. The rheogram showed that as the shear rate increased, the molecules in the polymers arranged themselves in the direction of flow, and thus the attraction between adjacent polymers decreased, and viscosity also decreased (Figure 1).

### 3.5. Determination of the Functional Properties of the Mucilage 

**Figure 2:**
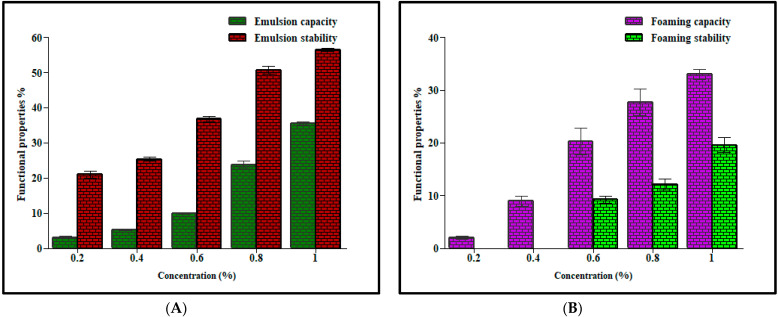
Comparison between emulsion capacity (%), emulsion stability (%) (**A**), foaming capacity (%) and foaming stability (%) (**B**) of *M. parviflora mucilage* obtained from the leaves (MLM) at different concentrations.

Functional properties of mucilage relied upon its emulsion capacity, emulsion stability, foaming capacity and foaming stability. The mucilage’s interfacial properties reduce the surface tension of water, where high emulsion capacity and emulsion stability indicate that the polymer can be used as a stabilizing agent in emulsions. Emulsion capacity and emulsion stability of MLM were found to be concentration-dependent, as shown in Figure 2A. As the concentration of mucilage increased, the entrapment of oil globules increased, and emulsion capacity and emulsion stability increased. The results of this study were comparable with previously reported values of some commercially available gums and mucilages. Hence it can be used as a stabilizer in oil in water emulsion [17]. The foaming properties are due to the ability of mucilage to increase viscosity and interfacial behavior [55]. The foaming capacity and foaming stability of MLM were also concentration-dependent, as shown in Figure 2B.

Meanwhile, MLM showed acceptable values for bulk density, tapped density, Carr’s index, Hausner ratio and angle of repose, as displayed in Table 2. Biomaterials with low bulk density show better disintegration as the particles will absorb more water by capillary action through the pores. Packing characteristics, such as flowability, compressibility and cohesiveness depend upon the Carr’s index and Hausner ratio, where MLM displayed Carr’s index and Hausner ratio values of 17.86% and 1.22%, respectively. Packing characteristics are good when Carr’s index and Hausner ratio are 12–18% and ˂1.25, respectively [24]. These findings suggest that MLM will show good packaging and disintegration properties when used in drug formulation. 

### 3.6. Morphological Analysis Using Scanning Electron Microscopy

**Figure 3:**
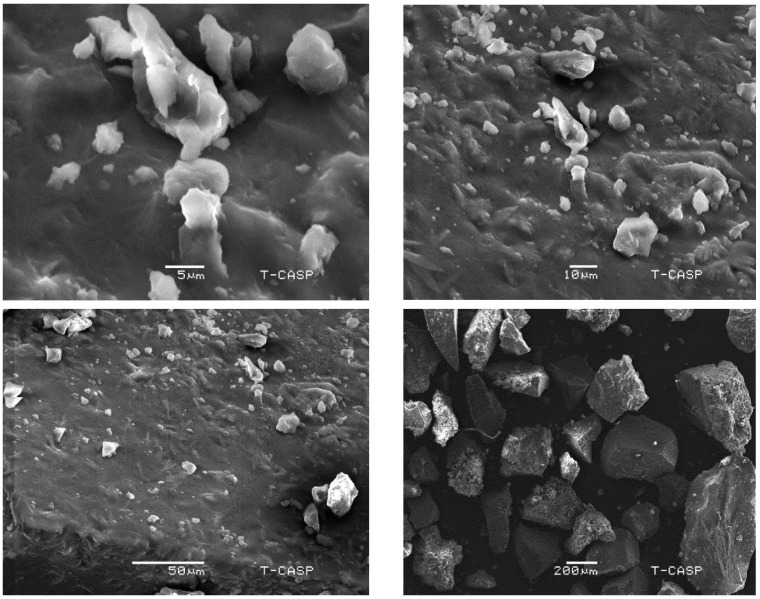
Scanning electron microscopy micrographs of *M. parviflora mucilage* obtained from the leaves (MLM) at different magnifications (2700×, 1000×, 500×, 100×).

The scanning electron microscope is an important instrument for powder characterization, i.e., size, shape, surface topography, texture, porosity, microstructure and agglomeration tendencies. Scanning the electron micrograph of MLM showed that particles are irregular, as they do not have fixed dimensions, and some of them are in the form of clusters; this, in turn, confirms the amorphous nature of MLM. Studies showed that the surface properties are greatly affected by the extraction, purification and preparation [18] (Figure 3).

### 3.7. Thermal Analysis Using Differential Scanning Calorimetry 

**Figure 4:**
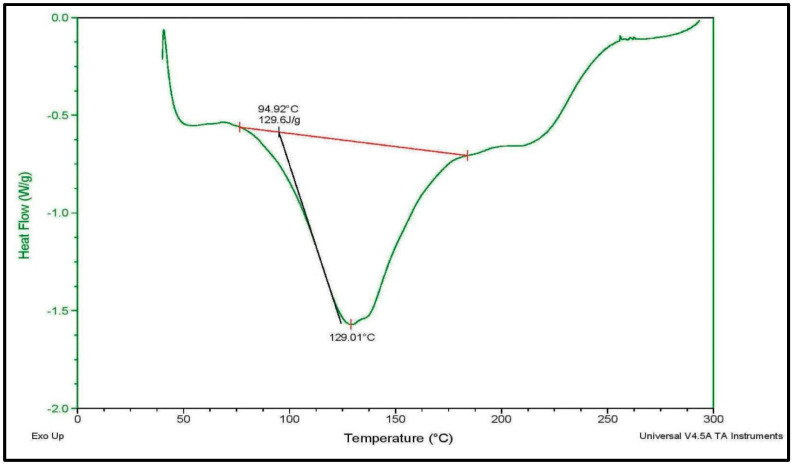
Differential scanning calorimetry (DSC) thermogram of *M. parviflora mucilage,* obtained from the leaves (MLM).

The DSC thermogram of MLM, displayed in Figure 4, represents a typical plot for polysaccharides. MLM displayed an early endothermic peak, appearing at 129 °C, that can be interpreted as losing the water that exists in the compound [56]. The endothermic peak below 150 °C makes natural polymers suitable to a wide class of therapeutic drugs [57]. The onset temperature was observed at 75 °C; meanwhile, the end state temperature was recorded at 185 °C. A pronounced melting peak was reported at around 129 °C, possessing an enthalpy value (∆H) of 129.6 J/g. The onset, the glass transition temperature and the melting peak are mainly attributed to the structural stability of the mucilages [58]. The lower branching manner within the structure leads to greater binding energy among the monosaccharide backbones, resulting in a higher value of (∆H) with a concomitant good thermal stability. From Figure 4, it is obvious that the thermal transition process of MLM occurs at a good enthalpy change (∆H), which in turn reflects the good thermal stability of MLM. 

### 3.8. Phytochemical Analysis of the Mucilage

**Figure 5:**
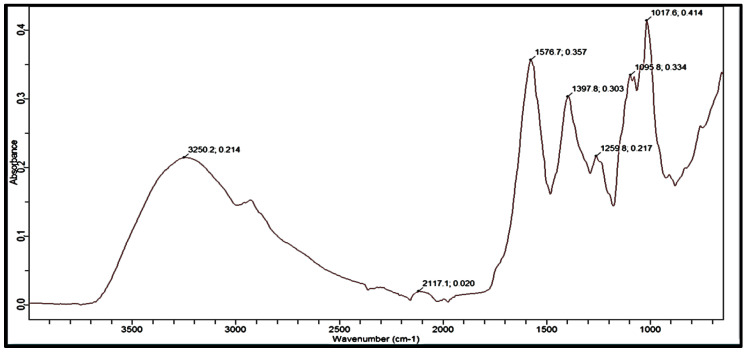
FTIR spectrum of M. *parviflora mucilage* obtained from the leaves (MLM).

The IR spectrum of MLM represented in Figure 5 revealed absorption bands at 3250 (cm^−1^) and 2850–2950 (cm^−1^), attributed to the stretching of the O-H and C-H bond, respectively. O-H stretching vibrations occur within a broad range of wavenumbers between 3500 and 3000 cm^−1^ and demonstrate several different features, i.e., the stretching bonds of free hydroxyl group and bonded O-H bands of carboxylic acid. The wavenumber range of 3000–2800 cm^−1^ could be assigned to C-H stretching, symmetric and asymmetric of free sugars. These peaks may also be due to the double overlapping with O-H [59]. These are the bands for the alkyl and hydroxyl functionality of carbohydrates. Peaks at 2117 (cm^−1^) indicate mono-substituted alkynes. FTIR spectra from the wavenumbers 1700–1500 cm^−1^ can be used to determine the structural properties of protein. Protein absorbance over these wavenumbers gives two absorption bands, amide I (1700–1600 cm^−1^) and amide II (1600–1500 cm^−1^). The amide II band is due to C-N stretching vibrations in combination with N-H bending [60]. The peak at 1576 (cm^−1^) represents the amide II band as well as the amine groups, where the amide II band is characteristic of proteins. Meanwhile, the peak at 1397 (cm^−1^) is mainly attributed to the presence of O-H bending and showed the existence of carboxylic acid moiety, where these peaks also represent the carboxylate ion stretches from uronic acid [61]. Peaks at 1259 (cm^−1^), 1095 (cm^−1^), and 1017 (cm^−1^) are due to aryl-O stretch or C-O stretching, showing the presence of aromatic ethers or alkyl-substituted ethers, respectively [62]. These findings suggest that MLM has hydroxyl, methyl, carboxyl groups and glycosidic bonds, which are the characteristic moieties of polysaccharides. Spectra obtained from MLM closely resembles already studied plant-derived mucilages [2].

**Figure 6:**
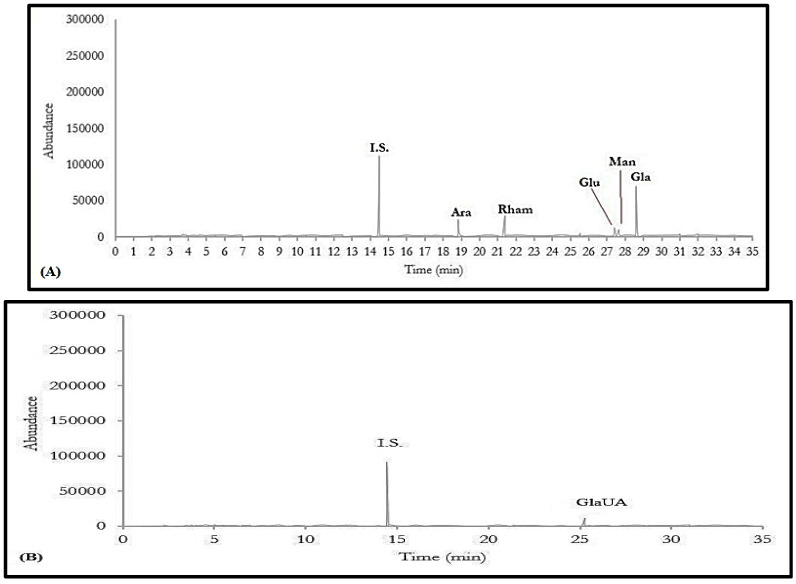
GC/MS chromatograms of neutral (**A**), and acidic (**B**) polysaccharides in *M. parviflora mucilage*, obtained from the leaves (MLM).

Moreover, GC/MS analysis of MLM revealed the presence of five neutral monosaccharides; namely, galactose, rhamnose, arabinose, glucose and mannose showing 51.09, 10.24, 8.90, 1.80 and 0.90 mg/g of MLM, respectively. Meanwhile galacturonic acid is the only detected acidic monosaccharide, forming 15.06 mg/g of MLM. The results of the GC/MS analysis of MLM are displayed in Figure 6, whereas GC/MS chromatograms for the standard neutral and acidic monosaccharides were displayed in the Supplementary Data (Figure S1). Additionally, the total phenolic content (TPC) of MLM was estimated to be 46.91 ± 1.12 µg equivalent of the gallic acid/mL of the mucilage sample containing 1 mg of MLM. Meanwhile, the total flavonoid content (TFC) of MLM was found to be 413.71 ± 0.52 µg equivalent of the rutin/mL of mucilage aqueous solution containing 100 mg of MLM, which, in turn, reflects the richness of the mucilage with polyphenolic contents that undoubtedly influence its antioxidant potential.

### 3.9. In Vitro Biological Evaluation of the Mucilage

#### 3.9.1. In Vitro Antioxidant Activity Assessment Using the 1,1-Diphenyl-2-picrylhydrazyl (DPPH*) Radical Scavenging Capacity Assay

**Figure 7:**
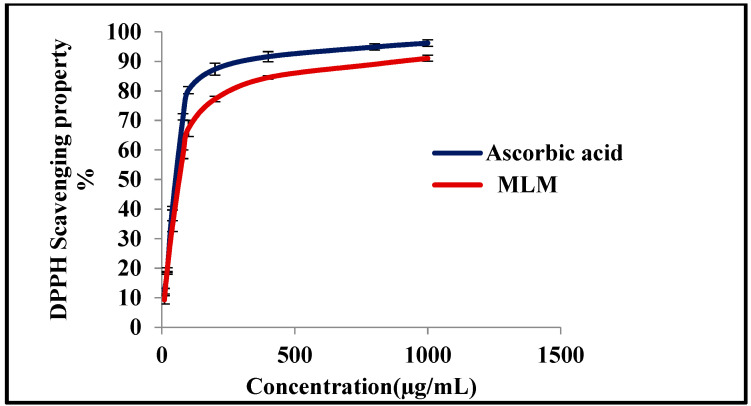
In vitro antioxidant activity assessment of MLM and compared to ascorbic acid using the 1,1-diphenyl-2-picrylhydrazyl (DPPH*) radical scavenging capacity assay.

Oxidative stress in humans damages the macromolecules comprising nucleic acid, lipids and protein, and results in cellular injuries. The human body has its mechanisms for defending against oxidative stress. However, in the cases of severe oxidative stress, the defensive mechanism fails to protect the human body. Thorough research has shown that hydroxyl radical and superoxide anion radicals play an important role in aging and carcinogenesis. Therefore antioxidants should be used to protect the body against these fatal damages. Natural antioxidants are preferred over synthetic antioxidants, as synthetic antioxidants cause toxicity and carcinogenesis. Thus, pharmaceutical and food industries mainly target the discovery of natural antioxidants [39]. DPPH assay is widely used to screen natural antioxidants, due to its accuracy and feasibility, where DPPH is a stable free radical that produces a violet color in ethanol. Antioxidants reduce DPPH by donating protons to it. As a result, its purple color fades rapidly [63]. The antioxidant activity of MLM was evaluated using different concentrations (10–1000 µg/mL) employing ascorbic acid as a control (Table 3). Results displayed in Figure 7 showed that MLM revealed a considerable antioxidant activity, where the antioxidant potential of MLM increases with an increase in concentration displaying IC_50_ value 154.27 µg/mL, whereas that of ascorbic acid was 41.21 µg/mL. Antioxidant activity of MLM might be due to the hydrogen donating capacity, and it is highly correlated to TPC and TFC, as it was previously reported that phenolic compounds and flavonoids existing in plants are responsible for their pronounced antioxidant activity [64].

#### 3.9.2. In Vitro Determination of DNA Damage Protective Potential of the Mucilage 

**Figure 8:**
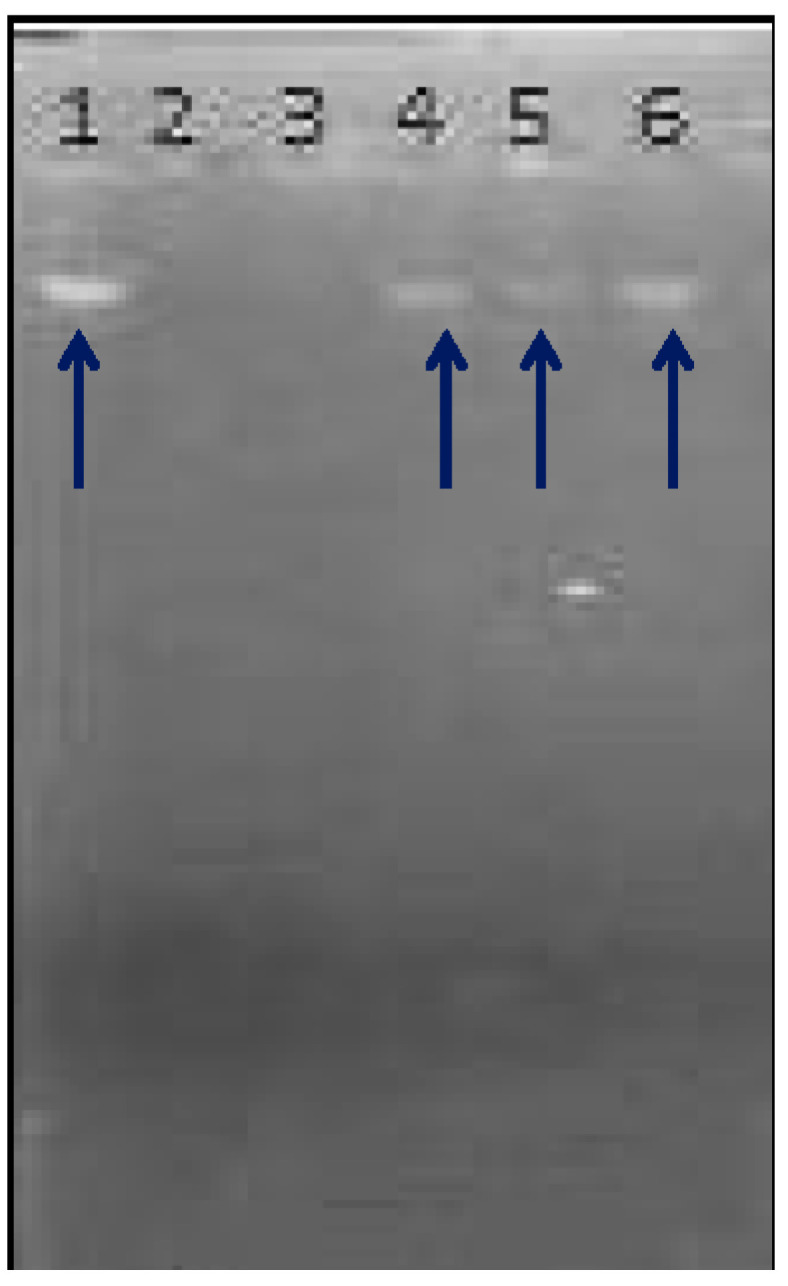
DNA damage protective effect of MLM. Lane 1: 4 µL DNA + 16 µL DW; **Lane 2**: 3 µL FR + 17 µL DW; **Lane 3**: 4 µL DNA + 3 µL FR + 13 µL DW; **Lane 4**: 4 µL DNA + 4 µL MLM (50 µg/mL) +3 µL FR + 9 µL DW; **Lane 5**: 4 µL DNA + 4 µL MLM (100 µg/mL) + 3 µL FR + 9 µL DW; **Lane 6**: 4 µL DNA + 4 µL MLM (200 µg/mL) + 3 µL FR+ 9 µL DW.

MLM was evaluated for its DNA damage protective ability where Fenton reagent was used as a DNA damaging reagent, as it can produce oxidative stress and destruct DNA. MLM protects DNA against oxidative damage induced by the Fenton reagent, as illustrated in Figure 8. The DNA band appears clearly in Lane 1 (DNA and distilled water), it completely disappears in Lane 2 (no DNA) and in Lane 3 (DNA with Fenton reagent that destroys DNA). Meanwhile, the DNA band began to reappear upon treatment with 50, 100 and 200 µg/mL of MLM in Lane 4–6, with a pronounced appearance in Lane 6, which contains the highest MLM concentration (200 µg/mL). MLM may inhibit the reaction of Fe^2+^ with hydrogen peroxide in the Fenton reagent, or it may donate the hydrogen atom or electron to inhibit the hydroxyl radicals [65]. 

#### 3.9.3. In Vitro Evaluation of Sun Protection Factor of the Mucilage

When skin is continuously exposed to UV radiation, free radicals are produced that oxidize the biological molecules, trigger oxidative damage and cause cancer. Hence, photoprotective agents are widely used to protect against UV radiation, where herbal skin protective agents are preferred over synthetic ones, being safer, less expensive and highly welcomed by a large category of people all over the globe [66]. Sun protection factor (SPF) is an indicator used for quantitative measurement of the efficacy of sunscreen products. Flavonoids and phenolic compounds present in the plant are more responsible for antioxidant and photoprotective properties, where phenolic compounds are highly correlated with SPF [67]. The MLM displayed pronounced SPF, estimated by 5.56 ± 0.15 and 10.93 ± 0.15 at 200 and 400 µg/mL, respectively. This significant SPF is attributed to the presence of polysaccharides, where previous studies reported that polysaccharides are a photoprotective agent and the richness of the MLM with polyphenolic compounds, as evidenced by its high total phenolic and total flavonoid contents [68,69]. These results suggest that MLM could be perfectly used in sunscreen formulation to protect skin against UV rays. The absorbance of 200 and 400 µg/mL of MLM at different wavelengths is illustrated in Table 4.

## 4. Limitations and Future Research

*Malva parviflora* is a seasonal herb in Pakistan with an optimum yield that varies according to season, location, climate and soil. MLM contains a moisture content of 5.84%; therefore, there is a risk of microbial contamination. Thus, there is a need to adopt analytical techniques to isolate bioactive polysaccharides from this mucilage. Research is needed to incorporate MLM in various drug delivery systems and to study the biocompatibility of MLM. However, further in vivo studies are highly recommended to confirm their applications before being used in preclinical trials and the pharmaceutical industry, in order to demonstrate the health benefits of MLM. In addition, the isolation of polysaccharides from complex matrices with protein requires effective and selective methods for pretreatment and extraction. The structure, chemical fingerprint and biological function of the mucilage are important to understand the structure–function relationship. Modification of the structure can be done by combining advanced analytical tools with sophisticated tools. Approval by food and drug regulatory authorities is mandatory to use MLM commercially. Regulatory authorities demand detailed toxicity and stability studies to approve mucilage and its products.

## 5. Conclusions

Herein, it was concluded that *M. parviflora* leaves contain a considerable amount of mucilage extracted in water, followed by the alcohol precipitation method. This mucilage consists of carbohydrates, proteins and amino acids, and it is free from toxic minerals. The extracted mucilage showed unique physicochemical, functional and micromeritic properties. The presence of the functional groups characteristic of polysaccharides was ascertained by the infrared (IR) and gas chromatography/mass spectrometry (GC/MS) analyses. GC revealed the presence of five neutral monosaccharides; namely, galactose, rhamnose, arabinose, glucose and mannose. Meanwhile, galacturonic acid is the only detected acidic monosaccharide. It showed noticeable antioxidant activity against the DPPH (1,1-diphenyl-2-picrylhydrazyl) radical. It also prevented oxidative damage to DNA caused by the Fenton reagent, as visualized in gel documentation system with considerable sun-protective properties. Hence, it can be used as a food supplement or as an ingredient in food and bio-industries; meanwhile, it can act as an attractive eco-friendly and sustainable biopolymer for the pharmaceutical industry. Further, it can be used as a natural antioxidant and protective therapy in skin diseases.

## Figures and Tables

**Table 1:** Physicochemical properties of *M. parviflora mucilage* obtained from the leaves (MLM).

Physicochemical Parameter	MLM
Solubility	Soluble in cold and hot water, less soluble in 1 M NaOH, HCl, citric acid and NaCl, and insoluble in all other organic solvents
pH (1% solution)	6.94 ± 0.02
Moisture content (%)	5.84 ± 0.03
Ash content (%)	15.60 ± 0.75
Water-soluble ash (%)	12.03 ± 0.55
Acid-insoluble ash (%)	0.87 ± 0.15
Swelling index	12.33 ± 2.51
Water-holding capacity (g/g)	2.57 ± 0.60
Oil-binding capacity (g/g)	2.03 ± 0.15

Results are expressed as Mean ± Standard deviation.

**Table 2:** Micromeritic properties of *M. parviflora mucilage* obtained from the leaves (MLM).

Property	MLM
Bulk density (g/cm^3^)	0.69 ± 0.06
Tapped density (g/cm^3^)	0.84 ± 0.05
Carr’s index (%)	17.86 ± 0.12
Hausner ratio	1.22 ± 0.12
Angle of repose	28.5 ± 0.5

Results are expressed as Mean ± Standard deviation.

**Table 3:** Percentage inhibition of 1,1-diphenyl-2-picrylhydrazyl (DPPH*) radical by ascorbic acid and MLM.

Concentration (µg/mL)	Ascorbic Acid	MLM
10	12.20 ± 0.96	9.29 ± 1.38
20	18.69 ± 0.20	19.05 ± 1.09
40	40.34 ± 0.61	34.26 ± 1.84
80	71.21 ± 1.07	58.54 ± 1.46
100	80.27 ± 1.24	67.63 ± 2.72
200	87.35 ± 2.00	77.26 ± 0.94
400	91.60 ± 1.70	84.54 ± 0.56
800	94.87 ± 1.0	89.03 ± 0.39
1000	96.20 ± 1.08	91.07 ± 1.00

Results are expressed as mean ± standard deviation.

**Table 4:** Absorbance of *M. parviflora mucilage* leaves (MLM) at concentrations of 200 and 400 µg/mL, at different wavelengths.

Wavelength (λ nm)	EE × I (Normalize)	MLM (200 µg/mL)	MLM (400 µg/mL)
290	0.0150	0.595 ± 0.01	1.071 ± 0.03
295	0.0817	0.587 ± 0.04	1.142 ± 0.11
300	0.2874	0.506 ± 0.18	1.062 ± 0.05
305	0.3278	0.574 ± 0.02	1.099 ± 0.16
310	0.1864	0.572 ± 0.05	1.106 ± 0.04
315	0.0839	0.569 ± 0.11	1.098 ± 0.02
320	0.0180	0.588 ± 0.03	1.091 ± 0.10

Results are expressed as mean ± standard deviation.

## Data Availability

Data supporting reported results may be found with the authors.

## Supplementary Materials

The following are available online at https://www.mdpi.com/article/10.3390/polym13234251/s1, Figure S1: GC/MS chromatograms for standard neutral and acidic monosaccharides.
