# Supplementary material for: Malva parviflora Leaves Mucilage: An Eco-Friendly and Sustainable Biopolymer with Antioxidant Properties
Source: Polymers (Basel). 2021 Dec 3;13(23):4251. doi: 10.3390/polym13234251 (PMC8659702; doi:10.3390/polym13234251)
Supplement: Supplementary file 1 [file polymers-13-04251-s001.zip › polymers-1461692-supplementary.pdf]

Malva parviflora **Leaves Mucilage: An Eco-friendly and Sustainable Biopolymer with Antioxidant Properties**

Supplementary materials

Figure S1: GC/MS chromatograms for standard neutral (A) and acidic (B) monosaccharides

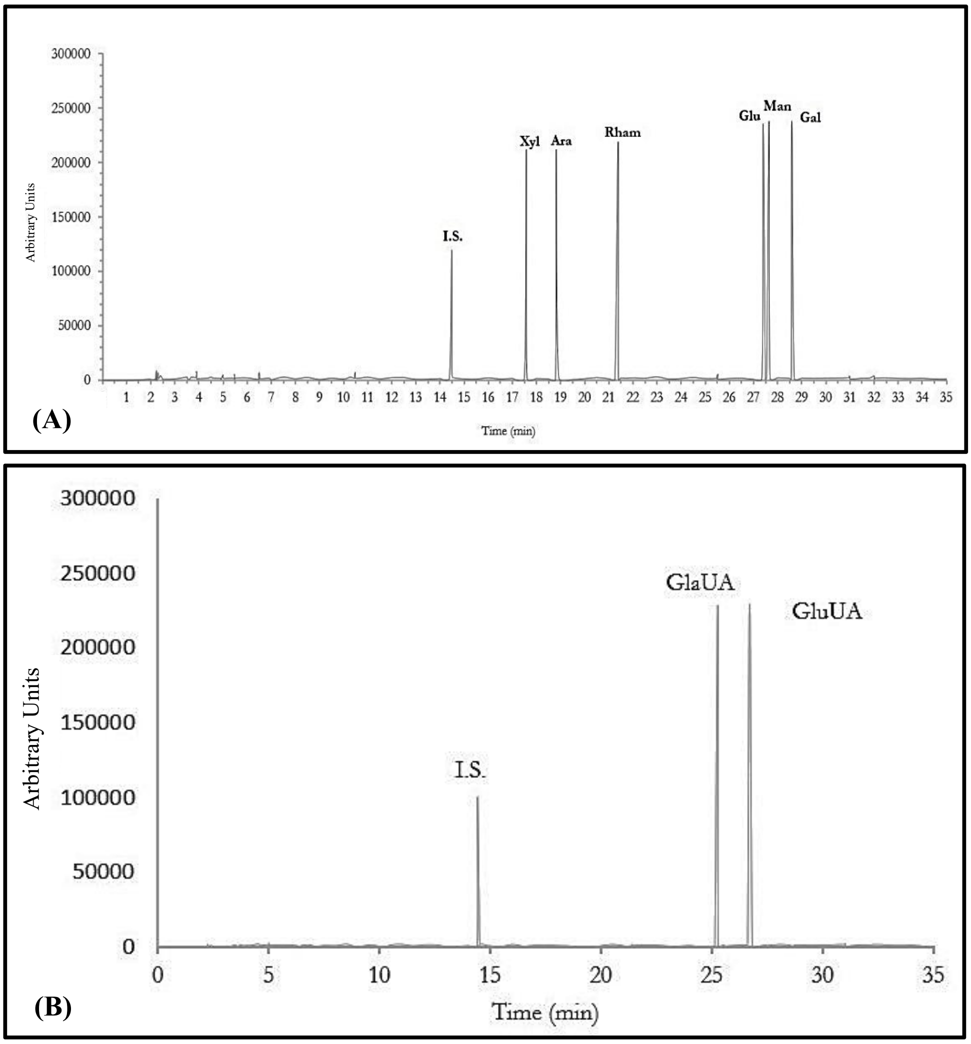

Figure S1: GC/MS chromatograms for standard neutral (A) and acidic (B) monosaccharides
